# Intra-articular injection in the hind limb joints of dromedary camels (*Camelus dromedarius*) using anatomical and arthrographic-guided landmarks

**DOI:** 10.14202/vetworld.2021.2055-2063

**Published:** 2021-08-10

**Authors:** Fahd Al-Sobayil, Madeh A. Sadan, Elsayed A. El-Shafaey, Jamal Allouch

**Affiliations:** 1 Department of Veterinary Medicine, College of Agriculture and Veterinary Medicine, Qassim University, Qassim, Saudi Arabia; 2 Department of Surgery, Anesthesiology and Radiology, Faculty of Veterinary Medicine, South Valley University, Qena 83523, Egypt; 3 Department of Surgery, Anesthesiology and Radiology, Faculty of Veterinary Medicine, Mansoura University, Mansoura 35516, Egypt; 4 Department of Anatomy, AL-Baath University, Syria

**Keywords:** anatomical, arthrographic, camels, hindlimb, intra-articular injection

## Abstract

**Background and Aim:**

A healthy joint is an important structure for the proper movement of the camel limb. Intra-articular (IA) injection is frequently used in veterinary practice for diagnostic and therapeutic purposes of joint injuries. Thus, the current study aimed to describe the injection of the hindlimb joints in dromedary camels based on the anatomical and arthrographic-guided landmarks.

**Materials and Methods:**

Eighteen orthopedically sound adult camels (mean±standard deviation age: 78±12 months) of both sexes were included in this study. Three camels were euthanized to identify anatomical features in the hindlimb joints and related structures. IA injections were performed in the hindlimbs of 5 camel cadavers to evaluate the optimal IA injection site, which was confirmed by arthrography. The optimized IA injection technique was applied in 10 live camels and confirmed by arthrocentesis and arthrography. For each joint, injection criteria (number of attempts, difficulty of injection, and successful injection) were assessed, scored, and statistically compared to the other joints.

**Results:**

The summation of IA injection criteria scores was significantly higher (p<0.05) in the femorotibial, femoropatellar, tibiotarsal, fetlock, pastern, and coffin joints in comparison to the hip joint.

**Conclusion:**

Anatomical and arthrographic-guided techniques offer considerable advantages for the characterization of anatomical landmarks and selection of the appropriate IA injection site in the hindlimb in dromedary camels. Furthermore, a reference approach for camels was established that is different from the approach for cattle and horses.

## Introduction

The worldwide population of dromedary camel (*Camelus dromedarius*) is approximately 14 million, distributed mainly in the Horn of Africa, the Middle East, and South Asia [1]. Dromedary camels are an important source of milk, meat, wool, and leather production and are also used for draught and riding purposes. Camel races are a popular sport in many countries and represent an important economic sector for income and tourism in these countries [2].

A healthy joint is an important structure for the proper movement of the camel limb. Intra-articular (IA) injection is frequently used in veterinary practice for diagnostic and therapeutic purposes and is increasingly used for the early diagnosis of joint injury [3-8]. This technique is easy to perform, cost-effective, and requires no special equipment [9]. Many reports have described hindlimb IA injection in horses [10-12], cattle [13], and small animals [14]. However, to the best of our knowledge, no comprehensive articles describe IA injection of the hindlimb in camels; hence, approaches described for horses or cattle have been widely applied to camels without verifiable data.

In the present study, we described the injection of the hindlimb joints in dromedary camels based on the anatomical and arthrographic-guided landmarks. A reference approach for the camel was established that is different from the approaches taken in cattle and horses.

## Materials and Methods

### Ethical approval

The study protocol was approved by the Committee of Animal Welfare and Ethics, Faculty of Agriculture and Veterinary Medicine, Qassim University, Saudi Arabia.

### Study period and location

The study was carried out from October 2019 to October 2020 in the Veterinary Teaching Hospital at the Qassim University Faculty of Veterinary Medicine, Saudi Arabia.

### Animals

Eighteen adult camels (Mean±standard deviation age: 78±12 months) of both sexes were included in this study. Camels were purchased from different localities of the Qassim Governorate, Saudi Arabia. All camels were clinically sound without a previous history of lameness or joint affection.

### Study design

The study was carried out in three parts, including anatomical, cadaveric, and *in vivo* studies. In the anatomical study, three freshly euthanized camels were used for the anatomical division of the hind limb joints. In the cadaveric study, five camel cadaver hindlimbs were used to evaluate the optimal site for IA injection and the injection site was confirmed by arthrography. In the *in vivo* study, ten live healthy adult camels were used to evaluate the *in vivo* accuracy of blind IA injections of the hindlimbs based on the cadaveric IA trials.

### Anatomical study

Three camels were randomly selected and euthanized using a high dose of rapid intravenous 30 mg/kg thiopental sodium (Thiowell 1%, Wellona Pharma 243, Surat, Gujarat, India). The anatomical landmarks of the hind limb joints and related structures for the correct IA injection were determined. The hindlimb cadavers were collected within 12 h of euthanasia. The specimens were moistened, wrapped in gauze, sealed in plastic bags, and stored at −20°C. For all investigations, limbs were thawed to room temperature, clipped, and cleaned. Anatomical landmarks of the hindlimb joints were evaluated and photographed by an expert anatomist (GA) with the aid of correlated anatomical references, for comparison to the corresponding arthrographic images. The hindlimb anatomical landmarks for the appropriate IA injection site were identified based on the anatomical features of each joint (articular surfaces, ligaments, synovial cavities, and the distance from the skin surface to the target joint).

### Cadaveric study

In the cadaveric study, IA injection sites for each hindlimb joint were shaved and thoroughly cleaned with warm water and soap. The limb was positioned in a suitable attitude for injection of each joint of the hindlimb. For all specimens (n=5), the injection of the hindlimb joint was performed by an experienced veterinary surgeon (EE). An adequate volume (10-50 mL, depending on the joint) of radiopaque iopamidol contrast agent (Scanlux®300, Sanochemia Pharmazeutika AG-Germania) was injected with minimal pressure into each joint, according to Alsobayil *et al*. [15]. Immediately after injection, the injected joint was fully flexed and extended 3 times. Subsequently, lateromedial (LM) and dorsoplantar (DP) radiographic projections of each joint were obtained using a Min X-ray HF 100/30 generator (Toshiba, Tokyo, Japan) with 70 kVp, 2.0 mAs, and 70 cm focal film distance. The operator considered the needle to be inserted into the joint space if the contrast agent injection met with little resistance, there was visible distension of the joint pouch, or the fluid could be retrieved following injection. The success of the injection was confirmed by the presence of contrast agent in the joint. The approach and landmarks for IA injection of each hindlimb joint were based on the following procedures:

### Coffin joint

**Figure-1:**
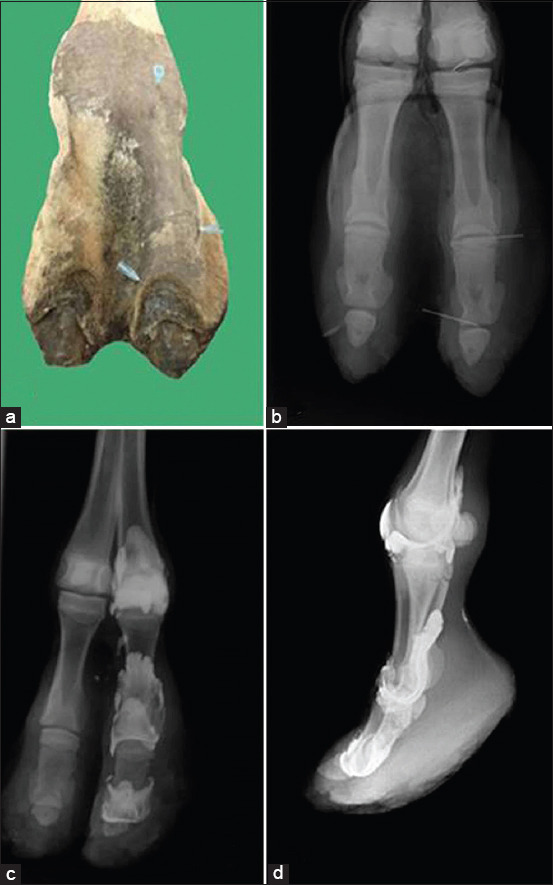
Sites for injection of the fetlock, pastern and coffin joints in a dromedary camel hind limb (a), dorsoplantar radiograph showing needle placement in the fore mentioned joints (b), dorsoplantar (c), and lateromedial (d) arthrograms of the fetlock, pastern, and coffin joints in the same camel.

IA injection of the coffin joint was performed approximately 5 cm medial or lateral to the vertical midline of the digit. The needle was inserted distally in the ventrolateral or ventromedial direction perpendicular to the bearing surface of the foot (Figure-1).

### Pastern joint

Injection of the pastern joint required continuous flexion and extension of the joint. The joint space was easily palpated with firm finger pressure just below the distal condyle of the first phalanx. The needle was inserted on the dorsal midline between the medial and lateral eminences on the distal end of the proximal phalanx and distally directed slightly medially or laterally (Figure-1).

### Fetlock joint

The needle was inserted at the midline through the dorsal aspect of the fetlock joint capsule at or slightly above the palpable joint space and perpendicular to the skin surface immediately proximal to the head of the 1^st^ phalanx. The needle went through the digital extensor tendon and was directed medially and parallel to the frontal plane of the joint to enter the dorsal pouch of the fetlock joint (Figure-1).

### Tarsal joint

**Figure-2:**
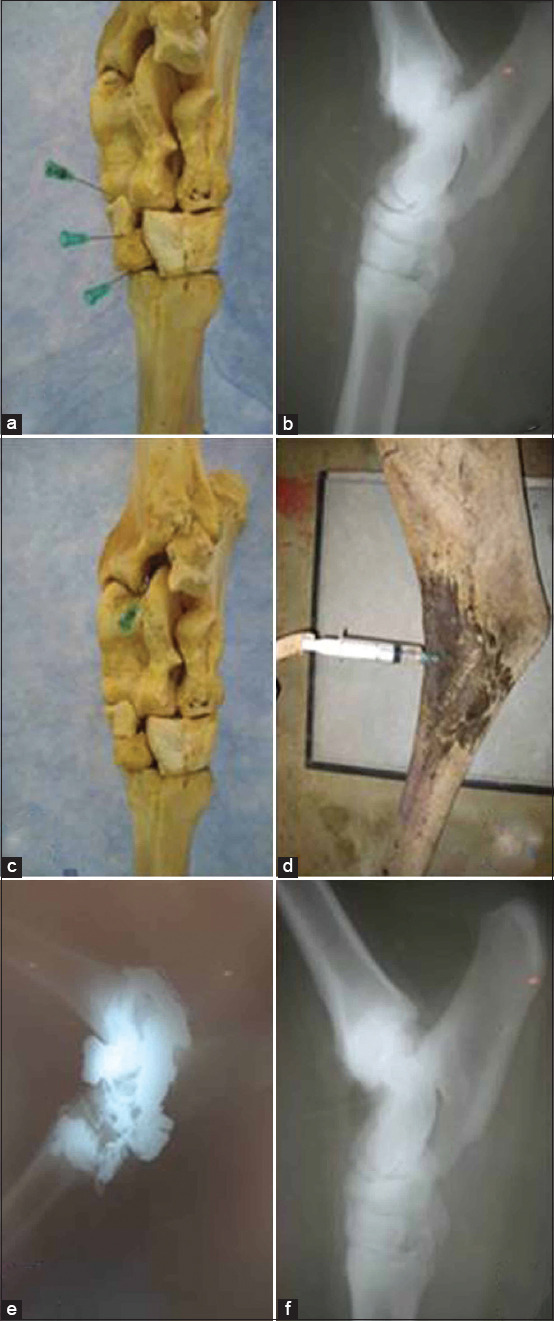
Sites for injection of the tarsometatarsal, distal intertarsal and proximal intertarsal joints in camel (a), Lateromedial (LM) radiograph showing needle placement in the fore mentioned joint (b), Site for injection of the tibiotarsal joint (c), Injection technique of the tibiotarsal joint in camel (d), LM arthrograms showing intra-articular presence of the contrast agent and needle after successful injection of the tarsal joint in camel (e and f).

The tarsal joint is composed of four joints: The tibiotarsal, proximal intertarsal, distal intertarsal, and tarsometatarsal joints (Figure-2a-d). Various approaches were tried for IA injection of the tarsal joint; the dorsal approach was the most feasible, especially with the tarsus extended (Figure-2). The tarsometatarsal joint was injected through dorsal, caudolateral, or caudomedial approaches. For the dorsal approach, the needle was inserted at the depression between the fused 2^nd^ and 3^rd^ tarsal bone and the 4^th^ tarsal bone. For the caudolateral or caudomedial approaches, the needle was inserted downward just proximal to the 4^th^ or the 2^nd^ metatarsal bone, respectively (Figure-2a and b). The dorsomedial approach was used for IA injection of the distal intertarsal joint. The needle was directed dorsally in a depression formed between the central and the 4^th^ tarsal bones or just distal to the central tarsal bone (Figures-2a and b). An alternative approach for the distal intertarsal joint involved an indirect injection through the connection with the tarsometatarsal joint. The proximal intertarsal joint was injected dorsolaterally (just below the distal end of the calcaneus) or dorsomedially (proximal to the central tarsal bone) (Figures-2a and b). In addition, dorsal injection of the tibiotarsal joint was achieved by inserting the needle ventrolateral to the tibial cochlea in a caudal and slightly proximal direction (Figures-2e and f).

### Stifle joint

**Figure-3:**
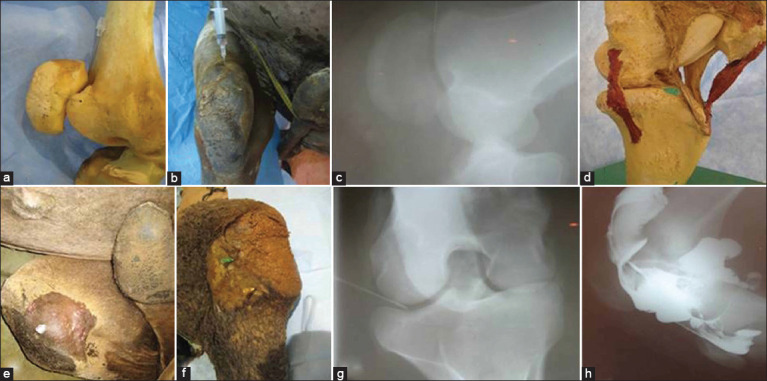
Site for injection of the femoro-patellar joint in camel (a), Injection technique of the forementiond joint in a live dromedary camel (b), Lateromedial (LM) radiograph showing correct needle placement for injection of the femoro-patellar joint (c), Site for injection of the femerotibial joint in camel (d), Injection technique of the forementiond joint in live dromedary camels (e and f), LM arthrograms showing intra-articular presence of the contrast agent and needle after successful injection of the stifle joint in camel (g and h).

The stifle joint consists of two joints, the femoropatellar joint and the femorotibial joint. For IA injection of the femoropatellar joint, the stifle joint was flexed and the dorsoproximal and distal ends of the patella and femur, respectively, were palpated. The needle was inserted distally between the patella and the femur in a slightly dorsal direction (Figures-3a-c). The femorotibial joint was injected dorsally from the lateral or medial sides (Figures-3d-f). For both sides, the patellar ligaments and the lateral or medial patellar retinaculum were palpated. In the dorsolateral approach, the needle was inserted between the palpable lateral patellar ligament and the lateral patellar retinaculum or just lateral to the lateral patellar retinaculum, directly above the palpable proximomedial edge of the tibia. In the dorsomedial approach, the needle was inserted between the medial patellar ligament and the medial patellar retinaculum or just medial to the medial patellar retinaculum above the palpable proximomedial edge of the tibia (Figures-3g and h).

### Hip joint

**Figure-4:**
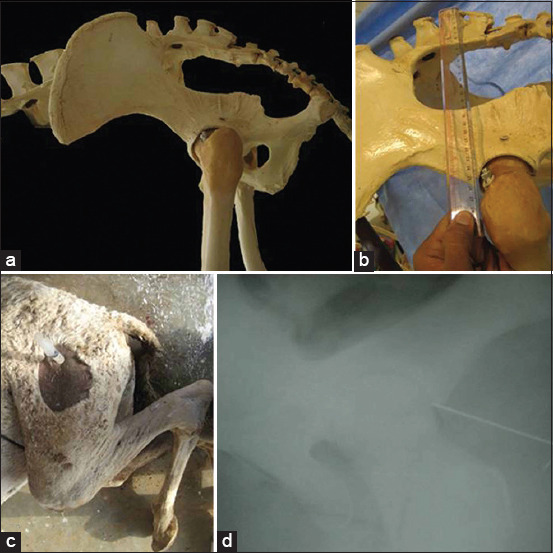
The anatomical intersection point for hip injection in camel (a and b), Injection technique of the hip joint in a dromedary camel (c), Lateromedial radiograph showing correct intr-articular placement of the needle for injection of the hip joint in camel (d).

The hip joint was blindly injected by inserting the needle at the intersection point above the palpable edge of the greater trochanter and directing the needle perpendicular to the vertebral column in the distomedial direction (Figures-4a and b). Positioning the camel in a lateral recumbent position and flexing the stifle joint was optimal for injection of the hip joint. This position facilitated the palpation of the greater trochanter, especially in thin camels (Figures-4c and d).

### *In vivo* study

To assess the reliability and accuracy of blind IA injections of the camel hindlimb, ten live healthy adult camels were used. The procedure was performed while the camels were sitting and sedated with xylazine hydrochloride (Seton 2%, Laboratorios Calier, S.A., Barcelona, Spain) at a dose of 0.2 mg/kg, IV. Each joint was aseptically prepared and injected blindly with needles of appropriate gauge and length.

### Evaluation parameters

Data for each IA injection were collected, including the number of attempts, difficulty of injection, and successful injection of each joint. Descriptive details of the scores and definitions of the hindlimb IA injection criteria are described inTable-1.

**Table 1:** Injection criteria scores for subjective assessment of the intraarticular injection of the hindlimb in dromedary camels.

Criteria	Score and description
Number of attempts	0 = Success with ≥ 3^rd^ attempt
	1 = Success with 2^nd^ attempt
	2 = Success with 1^st^ attempt
Difficulty of injection	0 = Difficult, several attempts with low confidence
	1 = Moderate, several attempts until successful injection
	2 = Easy, immediate and confident injection
Successful injection	0 = Poor, periarticular > 5mm from the target joint
	1 = Good, periarticular < 5mm from the target joint
	2 = Excellent, intraarticular of the target joint

### Statistical analysis

Statistical analysis was performed using the GraphPad Prism statistical software program (GraphPad Prism for Windows version 5.0, GraphPad Software Inc., USA). The IA injection criteria scores were compared among the hindlimb joints by the Kruskal–Wallis nonparametric ANOVA test. Differences were considered significant when p<0.05.

## Results

### Anatomical findings

Gross dissection was performed for each joint as part of the anatomical study. The skin and muscles related to each joint were carefully removed exposing the joint capsule. Gross anatomical dissection showed the numerous structures (articular structures, ligaments, blood vessels, and nerves) that were vulnerable to injury during needle insertion. Articular surfaces, ligaments, and synovial cavities were differentiated well (Figures-1-4). The mean distance from the skin surface to the target joint was about 3.0±0.25 cm and differed according to the site of the joint and its surrounding structures.

### Cadaveric findings

IA injection of the camel’s hindlimb was practical, reliable, and accurate. The anatomical landmarks determining the point of needle insertion for each joint were successfully identified in all cadavers and the joints were properly injected in all instances, as confirmed by arthrography (Figures-1-4). On plain and contrast arthrography, the anatomical features of each joint were readily distinguished. Each joint in the hindlimb of the dromedary camel could be precisely discriminated using anatomical and arthrographic-guided techniques.

The IA injection criteria scores were significantly higher (p<0.05) in the femorotibial, femoropatellar, tibiotarsal, fetlock, pastern, and coffin joints compared with the hip joint. The number of attempts and the difficulty of injection were significantly lower in the femorotibial, femoropatellar, tibiotarsal, fetlock, pastern, and coffin joints than in the other joints. The lowest successful injections were recorded for the hip joint compared with the other joints. The median and range of the criteria for IA injection of all camel hindlimbs are presented in Table-2.

**Table 2:** Median and range for subjective evaluation of injection criteria scores for intraarticular injection of the hindlimb in all investigated Dromedary camels.

Joint	Injection criteria (Median and range)		

	Number of attempts	Difficulty of injection	Successful injection
Coffin joint	0 (0-0)^a^	0 (0-0)^b^	0 (0-0)^c^
Pastern joint	0 (0-0)^a^	0 (0-0)^b^	0 (0-0)^c^
Fetlock joint	0 (0-0)^a^	0 (0-0)^b^	0 (0-0)^c^
Tarsal joint			
Tarsometatarsal joint	1 (1-2)^b^	1 (1-2)^b^	2 (1-2)^a^
Distal intertarsal joint	1 (1-2)^b^	1 (1-2)^b^	2 (1-2)^a^
Proximal intertarsal joint	1 (1-2)^b^	1 (1-2)^b^	2 (1-2)^a^
Tibiotarsal joint	2 (1-2)^a^	2 (2-2)^a^	2 (1-2)^a^
Stifle joint			
Femuropatellar joint	2 (1-2)^a^	2 (2-2)^a^	2 (1-2)^a^
Femurotibial joint	2 (1-2)^a^	2 (2-2)^a^	2 (1-2)^a^
Hip joint	0 (0-0)^a^	0 (0-0)^b^	1 (0-1)^a^

^a, b, c^: Medians and ranges with different superscript letters at the same column are significantly different at p<0.05

### Arthrographic findings

Arthrography of camel hindlimbs provided images with well-differentiated radiographic features of the synovial cavities for the evaluated joints (Figures-1-4). In addition, IA needle placement in the target joint and filling of the articular compartment with the contrast media was confirmed. For most of the hindlimb joints, a 20–22-gauge, 1-inch needle was optimal for IA injection. For the tibiotarsal, stifle, and hip joints, an 18-gauge, 1.5-inch needle was best. Descriptive details of the IA injection approaches and arthrographic features of the hindlimb joints on the DP and LM radiographs in dromedary camels are presented in Table-3.

**Table 3:** Intraarticular approaches and arthrographic features of the hindlimb joints on the dorsoplantar (DP) and lateromedial (LM) arthrograms in camels.

Joint	Intraarticular approach	Contrast volume (mL)	Needle		DP radiograph	LM radiograph

			Diameter (gauge)	Length (inch)		
Coffin joint	Dorsomedial/ dorsolateral	10 - 15	20 – 22	1	Large square joint with 2 short proximally oriented recesses.	It forms a large plantar sinus that extends to the middle of the 2^nd^ phalanx. The entire of coffin joint had small dorsal sinus.
Pastern joint	Dorsomedial/ dorsolateral	15 - 20	20 – 22	1	Large rectangular joint with 2 short axial and abaxial recesses extended distally.	It has a dorsal pouch and a long plantar pouch, extended to the middle of the 1^st^ phalanx. The dorsal pouch was narrower than the plantar one
Fetlock joint	Dorsal	20 - 25	20 – 22	1	It has 2 separate medial and lateral joint cavities. It appeared triangle in shape with its base directed distally and an apex directed proximally.	Appeared as a crescent with a relatively long plantar recess and short dorsal one.
Tarsal joint Tarsometatarsal joint	Dorsal/ Caudomedial/ caudolateral	10-15	20 – 22	1	The joint spaces between the various articulations of the tarsal joint (tarsocrural, talocalcaneal, intratarsal and tarsometatarsal joints) could be assessed.	Appeared large and irregular in shape with 2 cranial and caudal pouches. The caudal pouch is larger and longer than the cranial one. It extends proximally from the tibiotarsal joint and distally up to the tarsometatarsal joint. While, the cranial pouch extends only up to the proximal intertarsal joint.
Distal intertarsal joint	Dorsomedial	10-15	20 – 22	1		
Proximal intertarsal joint	Dorsomedial/ dorsolateral	10-15	20 – 22	1		
Tibiotarsal joint	Dorsal	25-35	18	1.5		
Stifle joint Femuropatellar joint	Dorsal	30 - 40	18	1.5	A small dorsal recess was detected and extended up to the proximal dorsal end of the tibia.	It has a large and small cranial and caudal pouch, respectively; both of them were extended along the whole length of the joint.
Femurotibial joint	Dorsomedial/ dorsolateral	35 - 50	18	1.5		
Hip joint	Lateral	25-30	18	1.5	The pelvis symphysis and coccygeal vertebrae are positioned in a straight line parallel to the midline. Both sides of the pelvic bones are symmetric.	Identified as the intersection point between 2 imaginary lines: a straight horizontal line starting from the ischium tuberosity and a vertical straight line starting from the root of the tail.

### *In vivo* findings

The IA injection technique for the hindlimb was well tolerated in live camels. No differences in the injection technique between *in vivo* and cadaveric studies were observed. Identification of the bony landmarks for IA injection of the hindlimbs was possible in all cases. Arthrocentesis was attempted before each injection. Then, the needle was advanced about 3–5 cm deep and limb retraction with resistance to injection was observed in two camels for the hip joint. In those animals, the needle was withdrawn and reinserted until positive arthrocentesis. Successful IA injection was achieved in all cases without any gross orthopedic abnormalities during or following the procedures. The average number of attempts required to inject the joint successfully was 1–3 and was higher in the hip joint.

## Discussion

A sound IA injection technique with a high success rate is essential for proper joint manipulation in farm animals [13,16]. Horse and cattle are often used as models for IA injection of large animal limbs. However, IA injection cannot be based on standard data from these animals and should be evaluated separately for the camel; there are methodological aspects of the camel that warrant further characterization. In addition, the occupation, anatomical variations in body size, height, and weight, and genetics of horses and cattle are different from camels and these differences can influence the correct limb injection [15,17]. Thus, the aim of this study was to investigate the efficacy of IA injection of the hindlimb joint using anatomical and arthrographic-guided landmarks, as an aid to clinicians performing arthrocentesis or injection.

To the best of our knowledge, this is the first study describing IA injection of the camel hindlimb. In the present study, no signs of articular injury were observed during injection of the hindlimb joints in live camels. Anatomical and arthrographic guidance facilitated the identification of anatomical landmarks, needle placement, and selectivity of the IA approach in camel hindlimbs. Similar findings were in agreement with previously published [9,18,19].

In the present study, a cadaveric model was employed to investigate IA injection techniques in the camel hindlimb. The accuracy rates for injection of the hindlimb joints in camel cadavers were as high as desired and were in agreement with previously published reports [15,20]. Moreover, there were no significant differences in IA injection success and techniques between fresh cadavers and live camels. The *in vivo* study was performed to exclude the possibility that the temperament, pain, and movement of the live camel during injection could change the results.

Contrast injection followed by radiography is a superior method for detecting extra-articular injection. Dissection depends on every plane of tissue being exactly exposed, whereas radiographic contrast is easily detected in a single radiograph [19]. In this study, arthrography of the hindlimb joints provided high-quality images in which well-differentiated features of the synovial cavities could be observed. In addition, arthrography was used to confirm successful injection through IA localization of the needle or contrast agent.

Several full flexions or extensions were essential for increasing the joint space to facilitate IA injection of the limb. Injections of hind limb joints in camels were easily performed after flexion, except for the tarsal joint. In the tarsal joint, extension facilitated injection and these findings were in accordance with the previous publications [12,21].

In this study, all IA injection approaches were successful and easy to perform with a low risk of articular cartilage damage. Scores for all injection criteria in the femorotibial, femoropatellar, tibiotarsal, fetlock, pastern, and coffin joints were higher compared with the injection criteria scores for the hip joint. The lower injection criteria score for the hip was due to the deep location of the joint beneath heavy muscle and well away from the lateral aspect of the proximal end of the femur. This location made the identification of landmarks difficult. These findings were in agreement with previously published studies [22,23].

In camels, IA injection of the coffin joint was performed with the needle inserted distally in the ventrolateral or ventromedial direction and perpendicular to the bearing surface of the foot. The optimal landmarks established in this study are similar to those previously established in the camel forelimb coffin joint [15,24,25]. However, in horses and cattle, the proper site for puncturing the coffin joint is proximal to the coronary band with the needle directed toward the extensor process of the 3^rd^ phalanx [22,26].

In horses, injection of the pastern joint can be performed by inserting the needle on the vertical midline above the proximal epicondyle of the second phalanx [7,10,21]. However, in camels, the joint space was easily palpated just below the distal condyle of the first phalanx. Thus, in camels, the optimal site for insertion of the needle was on the dorsal midline between the medial and lateral eminences on the distal end of the proximal phalanx.

Puncturing of the fetlock joint, in this study, was easily performed by the dorsal approach, especially with digit flexion. This was in agreement with a previous report [18]. However, the fetlock joint of horses can be entered using palmar/plantar and dorsal approaches [13,27].

In this study, the tarsal joint could be successfully injected utilizing several approaches. For selecting the proper clinical approach, reducing the time required and the necessity for potentially painful needle repositioning are important safety considerations for both the clinician and the camel. Injection of the distal intertarsal joint is technically more difficult than the tarsometatarsal joint because of its medial approach. Thus, this approach presents more danger to the clinician. The distal intertarsal joint can be simultaneously injected with the proximal joint through the tarsometatarsal joint because of the direct communication between the two joints. Moreover, the tibiotarsal joint is the easiest of all joints to inject because of its thin capsule and the obvious landmarks. The anatomical landmarks for a dorsal approach to joint injection are more distinct for the tibiotarsal joint, which may account for the reduced number of needle manipulations required for this approach. These observations were in agreement with previously published reports [28-32]. In horses, similar approaches were used for injection of the tarsal joint except for the distal intertarsal joint, which was injected laterally [33-35].

In horses, cranial and lateral approaches have been described for injection of the femoropatellar joint and the medial and lateral approaches have been described for injecting the femorotibial joint. However, in camels, we found that the optimal approach for the femoropatellar joint injection was dorsal to the stifle joint and the optimal approach for the femorotibial joint was dorsomedial or dorsolateral to the stifle joint [7,19].

In our study, the hip joint was blindly injected through a lateral approach by inserting the needle in the intersection point above the palpable edge of the greater trochanter and directing it perpendicular to the vertebral column in a distomedial direction. However, in horses, the dorsal approach is better for injection of the hip joint using blind or ultrasound-guided techniques [7,23,36].

Needle placement is a challenge for safe and effective IA injection techniques [9,12]. Needle placement is affected by several factors, including needle diameter and length and anatomical and radiographic features of the target joint. In camels, data to support a standard technique are lacking [15,18]. In this study, we found that a needle diameter and length of 20–22 gauges and 1 inch were appropriate for IA injection of most hindlimb joints. To ensure adequate needle localization, anatomical and arthrographic-guided landmarks were used. Similar recommendations were advised in the previous studies [20,37].

One challenging limitation in the IA injection of the camel limb is the evaluation of proper needle placement in relation to the target joint without complications. The criteria score system for evaluating injection techniques provided a simple tool for subjective assessment of the efficacy and success rates of IA injection techniques. These findings were in agreement with the previous studies [15,16].

IA injection of the camel limb is a relatively feasible and affordable radiation-free imaging tool that is easy to perform in skilled hands with minimal risks. IA injection is also cost-effective and requires no special equipment under field conditions. Moreover, IA injection can easily be incorporated into a diagnostic or therapeutic procedure of the joints. These findings are in agreement with previously published reports [19]. On the contrary, the use of blind IA injection has several limitations, including a needle insertion based on palpation of surface anatomic landmarks without visual control. This may result in incorrect needle placement and inadequate injection [23,36,37]. Therefore, further studies are necessary to evaluate other imaging-guided techniques versus blind ones to validate the effectiveness in clinical situations.

## Conclusion

Anatomical and arthrographic-guided techniques offer considerable advantages for the characterization of anatomical landmarks, needle placement, and selection of the appropriate site for IA injection of the hindlimb in dromedary camels. This study establishes a reference approach based on camel arthrography, which is different from cattle and horses. To the best of the authors’ knowledge, this is the first study of IA injection of the hindlimb in dromedary camels.

## Authors’ Contributions

FA, MAS, and EAE: Concept and designed the study. FA, MAS, EAE, and JA: Performed the experimental section. EAE and MAS: Analyzed and interpreted the data. All authors revised and approved the final manuscript.
